# PHOTOSTENT-02: porfimer sodium photodynamic therapy plus stenting versus stenting alone in patients with locally advanced or metastatic biliary tract cancer

**DOI:** 10.1136/esmoopen-2018-000379

**Published:** 2018-07-23

**Authors:** Stephen P Pereira, Mark Jitlal, Marian Duggan, Emma Lawrie, Sandy Beare, Pam O’Donoghue, Harpreet S Wasan, Juan W Valle, John Bridgewater

**Affiliations:** 1 The UCL Institute for Liver and Digestive Health, University College London, London, UK; 2 Department of Gastroenterology, University College London Hospitals NHS Foundation Trust, London, UK; 3 Cancer Research UK and University College London Cancer Trials Centre, London, UK; 4 Royal Free Hospital NHS Foundation Trust, London, UK; 5 Hammersmith Hospital, Imperial College Health Care Trust, London, UK; 6 Manchester Academic Health Sciences Centre, The Christie Hospital NHS Foundation Trust, The University of Manchester, Manchester, UK; 7 UCL Cancer Institute, University College London, London, UK

**Keywords:** cholangiocarcinoma, gallbladder, photodynamic therapy

## Abstract

**Background:**

Endobiliary stenting is standard practice for palliation of obstructive jaundice due to biliary tract cancer (BTC). Photodynamic therapy (PDT) may also improve biliary drainage and previous small studies suggested survival benefit.

**Aims:**

To assess the difference in outcome between patients with BTC undergoing palliative stenting plus PDT versus stenting alone.

**Methods:**

92 patients with confirmed locally advanced or metastatic BTC, ECOG performance status 0–3 and adequate biliary drainage were randomised (46 per group) to receive porfimer sodium PDT plus stenting or stenting alone. The primary end point was overall survival (OS). Toxicity and progression-free survival (PFS) were secondary end points. Treatment arms were well balanced for baseline factors and prior therapy.

**Results:**

No significant differences in grade 3–4 toxicities and no grade 3–4 adverse events due to PDT were observed. Thirteen (28%) PDT patients and 24 (52%) stent alone patients received subsequent palliative chemotherapy. After a median follow-up of 8.4 months, OS and PFS were worse in patients receiving PDT compared with stent alone group (OS median 6.2 vs 9.8 months (HR 1.56, 95% CI 1.00 to 2.43, p=0.048) and PFS median 3.4 vs 4.3 months (HR 1.43, 95% CI: 0.93 to 2.18, p=0.10), respectively).

**Conclusion:**

In patients with locally advanced or metastatic BTC, PDT was associated with worse outcome than stenting alone, explained only in part by the differences in chemotherapy treatments. We conclude that optimal stenting remains the treatment of choice for malignant biliary obstruction and the use of PDT for this indication cannot be recommended outside of clinical trials.

**Trial registration number:**

ISRCTN 87712758; EudraCT 2005-001173-96; UKCRN ID: 1461.

**Key questions**

**What is already known about this subject?**

- In patients with obstructive jaundice due to unresectable cholangiocarcinoma, small studies have suggested that photodynamic therapy (PDT) may improve biliary drainage and patient survival.

**What does this study add?**

- We conducted a large randomised controlled trial of porfimer sodium PDT in patients with confirmed locally advanced or metastatic biliary tract cancer.
- Patients undergoing PDT plus stenting had a worse outcome than those who underwent stenting alone.

**How might this impact on clinical practice?**

- We conclude that PDT for this indication cannot be recommended outside of clinical trials.

## Introduction

Cholangiocarcinoma and carcinoma of the gall bladder, collectively known as biliary tract cancer (BTC), are rare but highly aggressive malignancies associated with an unfavourable 5-year survival rate. Characterised by a relatively silent clinical course, they are often diagnosed at a late stage where curative resection can only be offered to <20% of patients.^1 2^

In advanced and metastatic disease, the mainstay of treatment is limited to palliative chemotherapy using a combination treatment of gemcitabine and cisplatin with improved overall survival (OS).^3^ Palliative endoscopic or percutaneous biliary tree stenting allowing tumour-related biliary tract drainage, is often required in order to avoid life-threatening biliary obstruction and subsequent infection in non-resectable disease. Despite these, the prognosis remains poor with a median survival of <6 months in patients with complex hilar lesions.^4^ Nevertheless, novel palliative approaches are needed to improve quality of life and survival in this patient group.

Photodynamic therapy (PDT) consists of endoscopically accessed localised delivery of light (most conveniently from a low-power, red laser) after prior systemic administration of a photosensitising agent, thereby initiating a focal, non-thermal, cytotoxic effect, tissue necrosis and apoptosis.^5^ PDT has been used for palliation in patients with unresectable cholangiocarcinoma, with uncontrolled studies reporting an improvement in cholestasis, quality of life and survival of patients compared with historical controls.^6 7^ These results have been supported by small randomised controlled trials which report a survival benefit for PDT with stenting over stenting alone for unresectable cholangiocarcinoma. Ortner *et al* randomised 39 patients to stenting with or without PDT and demonstrated a survival advantage of 493 vs 98 days in favour of PDT.^8^ The patients in this study were those in whom jaundice could not be relieved by stenting, so it remains unclear whether PDT may also improve the survival of the majority of patients whose cholestasis can be relieved by biliary stenting. Zoepf *et al* randomised 32 patients to stenting with or without PDT and demonstrated a survival advantage of 21 vs 7 months in favour of PDT.^9^ These studies were small and in keeping with the standard of care at the time, few patients received palliative chemotherapy. Adverse events (AEs) related to PDT were minor and there was no early mortality. Cheon *et al* reported a retrospective analysis of 232 patients with cholangiocarcinoma, where the outcome in patients receiving PDT and endoscopic biliary drainage versus drainage alone was compared. Improved survival was observed in the PDT group with a median survival of 9.8 months compared with 7.3 months in the stent alone group. Moreover, longer stent patency time was observed following PDT.^10^ Reports of other non-randomised trials exist in the literature. A meta-analysis which included 531 subjects in five different trials (out of which 230 received PDT), reported improved survival after PDT compared with stenting alone.^11^ Lastly, in a three-centre, single-arm phase II trial of 36 patients with locally advanced BTC, we showed that PDT was associated with a low toxicity profile and a median survival of 12 months.^12^

Given these encouraging reports, a UK multicentre, randomised, phase III study was designed to assess the efficacy and safety of porfimer sodium PDT with biliary stenting versus biliary stenting alone in advanced or metastatic BTC.

## Materials and methods

### Design

This was a multicentre, open-label randomised controlled phase III trial, designed and developed by the PHOTOSTENT-02 Trial Management Group under the auspices of the UK National Cancer Research Institute Upper Gastrointestinal Cancer Clinical Studies Group. The study was sponsored by the University College London and coordinated by the Cancer Research UK and University College London Cancer Trials Centre. Regulatory approvals were obtained, and all patients gave written informed consent. The trial was run in accordance with the Declaration of Helsinki. An Independent Data Monitoring Committee regularly reviewed the data on safety and efficacy.

### Patients

Patients were eligible if they had a histopathological or cytological diagnosis of non-resectable, recurrent or metastatic biliary tract carcinoma (intrahepatic or extrahepatic cholangiocarcinoma or gallbladder carcinoma); had an ECOG performance status of 0, 1, 2 or 3; were aged ≥18 years and had an estimated life expectancy of >3 months. Patients were also required to have adequate biliary drainage before randomisation, with no evidence of active uncontrolled infection. Patients with highly suspicious findings for malignancy were permitted following central review and study entry was based on a case-by-case decision. Patients who had undergone non-curative surgery, had received prior radiotherapy and chemotherapy were eligible.

Patients were excluded if they had received treatment with curative intent for current disease (ie, a prior resection, radical radiotherapy or chemotherapy) within the last 12 weeks; or palliative chemotherapy or radiotherapy within the previous 4 weeks. If they had any of these prior treatments, there had to have been clear evidence of disease progression prior to inclusion. Additional exclusion criteria included a history of prior malignancy that could interfere with response evaluation, porphyria, pregnancy or breast feeding or lack of informed consent. Once the eligibility and exclusion criteria were checked at the registration telephone to the trial centre, patients were randomised to either PDT+stenting or stenting alone. Treatment allocation was given during the call and subsequently confirmed via fax and sent together with the case report forms. Randomisation used a minimisation algorithm, stratified for primary tumour site, disease extent (locally advanced vs metastatic), performance status, type of prior therapy and recruiting centre.

### Study entry

The initial patient evaluation included a history and physical examination, laboratory studies (complete blood count, biochemistry panel including liver biochemistry and tumour markers) and chest X-ray. In women of childbearing age, a negative serum pregnancy test was required. If not already done within the previous 4 weeks, tumour staging was performed by CT or MRI/cholangiopancreatography (MRI/MRCP) and diagnosis confirmed by endoscopic brush cytology or biopsy. If cytology or histology were negative at the first endoscopy, a repeat intervention (endoscopic retrograde cholangiopancreatography (ERCP), CT-guided or ultrasound-guided percutaneous needle biopsy or endoscopic ultrasound-guided fine-needle aspiration) was performed.

### Treatment

For entry into the study, all patients in both arms required adequate endoscopic or percutaneous biliary drainage and insertion of endoprosthesis into the right and left intrahepatic biliary tree up to 4 weeks before randomisation. The placement of endoprosthesis was defined as technically successful when the stent bridged the main stricture to the right and/or left hepatic ducts ensuring sufficient passage of contrast medium through the stent into the duodenum (endoscopic plastic endoprosthesis, 7–10 French (F) diameter, Cotton-Huibregtse type, Cook Ireland, Limerick). Oral ciprofloxacin was given before the ERCP and continued for at least 24 hours according to local hospital guidelines. Prior placement of uncovered metal stents was not an exclusion criterion.

Once adequate biliary drainage had been achieved, patients randomised to stenting alone did not undergo repeat stenting unless clinically indicated. Those randomised to PDT and stenting received intravenous porfimer sodium (Photofrin; Axcan Pharma, Mont Saint-Hilaire, Canada) at a dose of 2 mg/kg bodyweight. At ERCP 48 hours later, the previously placed endoprostheses were removed, and endoluminal photoactivation performed either through a clear 10 F Huibregtse-Cotton endoscopic catheter introduced proximally above the strictures, or directly by inserting the laser quartz fibre (Medlight SA, Ecublens, Switzerland; 400 μm core diameter, 20–50 mm cylindrical diffuser tip with an X-ray marker on both ends of the diffuser) directly across the stricture.

Photoactivation was performed at 630–635 nm using a light from a diode laser (Diomed, Cambridge, UK), with a linear diffuser exit dose of 186 J/cm, at linear irradiance of 300 mW/cm. During the procedure, all patients received oxygen via a nasal catheter and conscious intravenous sedation with midazolam and fentanyl. A new set of endoprosthesis was inserted after the completion of treatment.

Patients remained on the ward in subdued lighting after administration of the photosensitiser, followed by re-adaptation to indirect sunlight for increasing periods during the morning and late afternoon of each day. Bright indoor light was permitted after the initial 2–3 days period. Patients were also given written and oral advice to avoid direct sunlight for 1–2 months. Patients randomised to stent alone were optimally stented, if necessary then proceeded to surveillance.

### Assessment during and after treatment

In the PDT+stenting group, clinical and laboratory assessments, including full blood count, urea and electrolytes, liver function tests, ECOG performance status, weight and clinical examination, were repeated at 7 and 28 days after light activation. Thereafter, patients in both trial groups were followed up at 3 monthly intervals. Formal tumour response evaluation by CT or MRI/MRCP was performed at 28 days and again at 6 and 12 months following administration of porfimer sodium.

Tumour response, by RECIST 1.0,^13^ was assessed radiologically (CT or MRI) at 3 and 6 months (post hoc analysis). Tumour control was defined as either complete response, partial response or stable disease; progressive disease was defined as either objective tumour progression or the confirmed emergence of local non-primary, metastatic or nodal disease. After study treatment had finished, patients were reviewed in clinic every 3 months until disease progression. Follow-up visits consisted of clinical assessment and either a CT or MRI scan to assess tumour status; once progressive disease was documented, patients were followed up for survival only.

Endoprostheses exchange was performed at 6 monthly intervals or earlier if clinically indicated. In some patients with evidence of tumour progression at follow-up CT/MRI or ERCP, a second PDT was allowed at least 6 months after initial treatment. During the study period, patients could receive chemotherapeutic agents after day 28 at the discretion of their treating oncologist.

### Assessment of adverse events and quality of life

Treatment-related toxicity was assessed at discharge and at each subsequent follow-up visit. All AEs were graded according to the National Cancer Institute Common Toxicity Criteria (V.3.0). The frequency of grade 3 and 4 AEs has been reported by summarising the maximum grade experienced by each patient. Because patients were permitted to have abnormal liver biochemistry as long as adequate biliary drainage was achieved before randomisation, grade 3/4 liver biochemical abnormalities at day 7 and 28 were not regarded as AEs if they had been present before treatment.

### Statistical considerations

The primary outcome was OS and the secondary outcomes were progression-free survival (PFS) and AEs. The trial was designed to detect an increase in median survival of 4 months (or more), from 8 months in patients not receiving PDT to 12 months in those who did receive PDT (which was the lowest median survival in previous studies.^7–9^ To detect this difference in survival the trial required 240 patients to complete the study. This was based on 80% power, 5% statistical significance (two-sided) and assumed that the trial would recruit for up to 3 years with at least 12 months follow-up. Patients were randomised by telephoning the Cancer Research UK and University College London Cancer Trials Centre, which coordinated the trial.

All analyses were done on an intention-to-treat basis. The treatment difference of AEs was assessed by the test of proportions. OS was calculated from date of randomisation until date of death. PFS was measured from randomisation until date of progression or death. Patients not having an event reported were censored at the date of their last follow-up. OS and PFS were analysed using Kaplan-Meier curves and Cox proportional hazards model was used to estimate the HR, comparing PDT versus stent alone. All analyses were performed using Stata V.12.1 (StataCorp, College Station, Texas, USA).

## Results

### Patient characteristics

**Figure 1:**
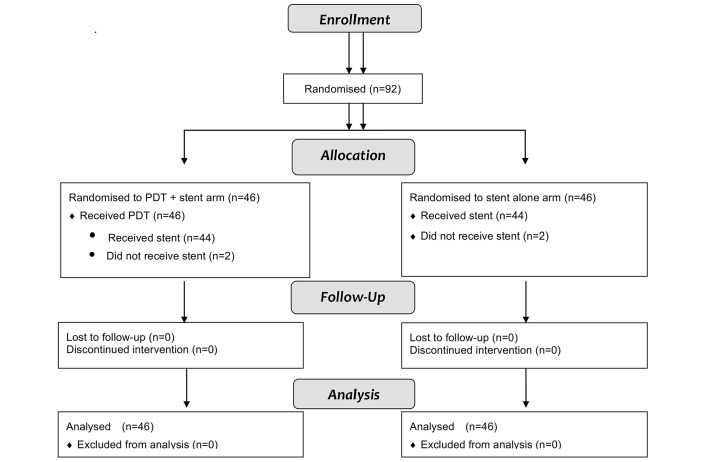
Consolidated Standards of Reporting Trials 2010 flow diagram of patients in the PHOTOSTENT-02. PDT, photodynamic therapy.

Figure 1 summarises the progression of patients through the trial; over a 28-month period, 92 patients were recruited (46 per group) from eight centres. There were 55 males and 37 females, with a median age of 67.4 (range 25.4–85.4) years. The two groups were well balanced for age, gender, disease stage and site, performance status and history of prior chemotherapy (table 1).

**Table 1:** Baseline characteristics of trial patients in the PHOTOSTENT-02 trial

	PDT+stenting n=46 N (%)	Stenting alone n=46 N (%)
Sex		
Male	29 (63)	26 (57)
Female	17 (37)	20 (43)
Disease status		
Locally advanced	32 (70)	34 (74)
Metastatic	14 (30)	12 (26)
Primary tumour site		
Gallbladder	3 (7)	5 (11)
Bile duct	43 (93)	41 (89)
Prior therapy		
None	36 (78)	37 (80)
Chemotherapy	7 (15)	7 (15)
Other	3 (7)	2 (4)
ECOG performance status		
0	15 (33)	14 (30)
1	21 (46)	20 (43)
2	9 (20)	10 (22)
3	1 (2)	2 (4)
Age (years), median (IQR)	67.7 (62.8–72.8)	67.3 (60.4–74.2)

Recruitment was stopped on 18 December 2009 on advice of the Independent Data Monitoring Committee, after 92 patients had been recruited, and there had been a persistent difference in OS between the trial groups, in which those receiving PDT had worse survival. Follow-up data were collected on all patients entered into the trial until June 2011, when the database was closed for analysis. The five top recruiting sites then underwent independent quality assurance audit and monitoring to confirm adherence to the protocol and Good Clinical Practice, and to verify the data. The Cancer Trials Centre also underwent an independent quality assurance audit.

### Treatment compliance

All patients allocated to receive PDT did so, and the median time from randomisation to PDT treatment was 9 (range 5–12) days (table 2). PDT was technically successful in all patients, with light delivery performed after a median drug light interval of 48 (IQR range: 44–48) hours. During follow-up, 41% and 54% of patients required repeat stenting in the PDT and stenting only arms, respectively, at a median of 2–3 months after randomisation. Four patients did not have a stent as per protocol—two per arm (figure 1).

**Table 2:** Treatment details of patients in the PHOTOSTENT-02 trial

	PDT+stenting	Stenting alone	P values
	n=46 N (%)	n=46 N (%)	
Time from randomisation to PDT (days)*	9 (5–12)	NA	
Patient underwent repeat biliary stenting			
No	27 (59)	21 (46)	0.21
Yes	19 (41)	25 (54)	
Number of separate restenting procedures			
1	9 (47)	11 (44)	0.55
2	6 (32)	6 (24)	
>3	4 (22)	8 (32)	
Time from randomisation to first restenting procedure (days)*	96 (74–139)	69 (46–134)	0.29
Additional treatments received			
Received chemotherapy	13 (28)	24 (52)	0.019
Of which:			
Gemcitabine	3	8	
Gemcitabine+cisplatin	6	12	
Other chemotherapy regimen	4	4	
Time from randomisation until starting chemotherapy (days)*	135 (65–297)	43 (29–106)	0.005
Other additional treatment	5 (11)	14 (30)	0.02
Of which:			
Radiotherapy	2	3	
Other †	3	11	

*Median (IQR). The Mann-Whitney U test was used to compare median time to event for only those patients who underwent repeat biliary stenting or received chemotherapy.

†Stent alone (11 patients, 14 records): ERCP (2); percutaneous transhepatic drainage (1); cancer bowel operation (1); percutaneous drainage of ruptured liver abscess (1); ascites drainage (1); palliative care (1); palliative gastrojejeunostomy (1); HDAC inhibitor (1); catheter inserted (1); nutritional support (1); antibiotics for cholangitis (1); bile acid sequestrant (1); antibiotics for chest infection (1). PDT (3 patients, 3 records): peritoneum transhepatic stent (1); palliative care (1); gentamycin and oral ciprofloxacin for sepsis (1).

ERCP, endoscopic retrograde cholangiopancreatography; HDAC, histone deacetylase; PDT, photodynamic therapy.

### Survival and disease progression

**Figure 2:**
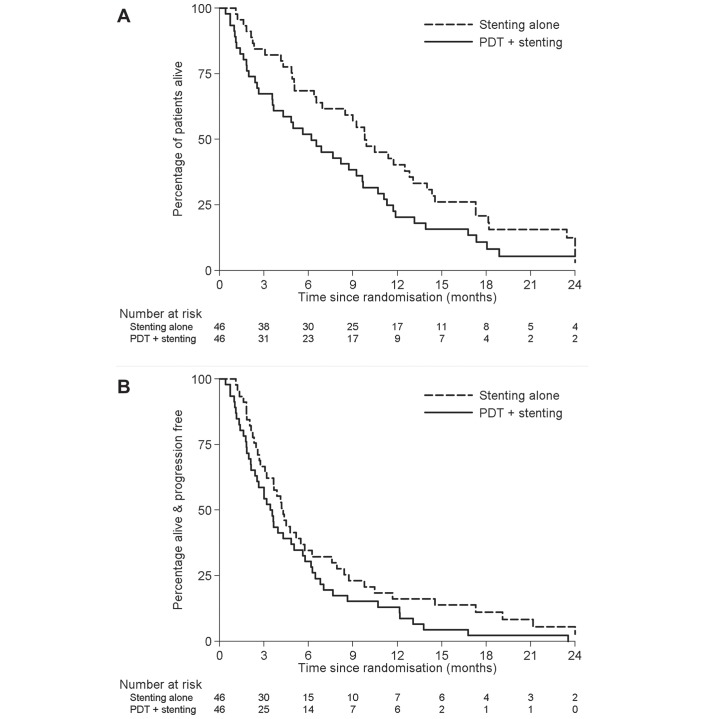
Kaplan-Meier estimates of (A) overall survival and (B) progression-free survival by treatment arm. (A) Median survival in the photodynamic therapy (PDT) and stent alone arms are 6.2 months (95% CI 3.5 to 9.2 months) and 9.8 months (95% CI 6.5 to 12.8 months), HR 1.56 (95% CI 1.00 to 2.43), p=0.048. (B) Median progression-free survival in the PDT and stent alone arms are 3.4 months (95% CI 2.1 to 5.0 months) and 4.3 months (95% CI 3.1 to 5.7 months), HR 1.43 (95% CI 0.93 to 2.18), p=0.10.

At the time of the final analysis, median follow-up was 7.7 months and 82 patients had died (76 from disease progression). There was one treatment-related death in the PDT arm secondary to a gallbladder empyema. OS was inferior in the PDT arm compared with stenting alone (median survival: 6.2 vs 9.8 months (HR 1.56, 95% CI 1.00 to 2.43, p=0.048); figure 2A). After adjusting for the randomisation stratification factors, the HR was 1.83, 95% CI 1.13 to 2.96, p=0.014. The survival rate in the PDT arm at 12 months was half that of the stent alone arm (20.3% vs 40.3% survival).

Forty-two (91%) and 46 (100%) of patients progressed or died in the stenting alone and PDT+stenting arms, respectively. There was weaker evidence of decreased PFS among the PDT patients (HR 1.43, 95% CI 0.93 to 2.18, p=0.10; median PFS 3.4 vs 4.3 months; figure 2B); adjusting for stratification factors, HR 1.61 (95% CI 1.02 to 2.54), p=0.042.

### Effect of chemotherapy on survival

Due to the unexpected adverse effect of PDT on survival, we investigated the possible reasons for this. Patients were permitted to have other therapies at least 28 days after randomisation (to allow for resolution of any AEs). A higher proportion of patients in the stent alone group received subsequent chemotherapy (24 patients (52%) vs 13 patients (28%); p=0.02) and those who received it did so more rapidly following randomisation (median time to start of chemotherapy: 1.4 vs 4.4 months; p=0.005). We observed that receiving chemotherapy after randomisation was associated with a significantly improved survival. After adjusting for trial arm and baseline stratification factors, patients receiving chemotherapy were 85% less likely to die (HR 0.15 (95% CI 0.07 to 0.29), p<0.001. Median survival in patients who went on to receive chemotherapy was 13.0 vs 4.1 months for those who did not. Table 2 details that 50% of such patients received cisplatin and gemcitabine, the international standard for advanced disease. Another 25% received gemcitabine and 25% received another regimen. The impact of a differing chemotherapy regimen on survival is unlikely to have affected the outcome, in particular mortality within 3 months of randomisation.

When comparing the OS between the two trial groups, adjusting for whether patients had chemotherapy or not after randomisation reduced the HR from 1.56 to 1.39 (95% CI 0.89 to 2.17, p=0.15), that is, an approximate 30% reduction. However, this factor did not fully explain the survival difference associated with PDT.

### Adverse events

PDT was generally well tolerated; apart from liver toxicities, there was only one grade 4 toxicity (sepsis) in the first month and one death secondary to empyema in the PDT arm. There was a single episode of ERCP-induced pancreatitis but no episodes of major bleeding or photosensitivity reactions in the PDT group. No suspected unexpected serious adverse reactions were reported.

There were 39 patients (85%) with any grade 3–4 AE in the PDT arm compared with 30 (65%) in the stenting only group (p=0.030), which was partly due to a difference in bilirubin (37% (PDT+stent) vs 20% (stent alone); p=0.06; table 3). There were 36 patients with grade 3–4 liver function (bilirubin, alkaline phosphatase, alanine transaminase and gamma-glutamyltransferase) abnormality in the PDT arm compared with 28 in the stent only arm, the median survival of whom are 5.0 and 9.9 months, respectively (log-rank p=0.017) suggesting a detrimental effect of PDT on liver function that is associated with early death. There were more early deaths (within the first 3 months) in the PDT arm compared with stent only among those patients with any liver toxicity during treatment (PDT: 39% (14/36); stent: 18% (5/28); p=0.068).

**Table 3:** Grade 3–4 adverse events during treatment in the PHOTOSTENT-02 trial

Adverse event	PDT+stenting n=46 N (%)	Stenting alone n=46 N (%)	P values
Gamma-glutamyltransferase	28 (61)	22 (48)	0.21
Alkaline phosphatase	22 (48)	17 (37)	0.29
Bilirubin	17 (37)	9 (20)	0.064
Alanine transaminase	4 (9)	6 (13)	0.50
Anaemia	3 (7)	2 (4)	0.65
Albumin	3 (7)	2 (4)	0.65
Biliary sepsis	2 (4)	1 (2)	0.56
Pancreatitis	1 (2)	0 (0)	0.32
Photosensitivity	0 (0)	0 (0)	–
Fever	0 (0)	0 (0)	–
Other toxicity	9 (20)	2 (4)	0.024
Fatigue	2 (4)	0 (0)	
Ascites/abdominal bloating	2 (4)	0 (0)	
Prothrombin time	2 (4)	0 (0)	
Nausea	2 (4)	0 (0)	
Sepsis/septicaemia	1 (1)	1 (1)	
Cholangitis	1 (1)	0 (0)	
Vomiting	0 (0)	1 (1)	
Abdominal pain	1 (1)	0 (0)	
Urinary retention	0 (0)	1 (1)	
Constipation	1 (1)	0 (0)	
Insomnia	1 (1)	0 (0)	
Somnolence	1 (1)	0 (0)	
Any toxicity	39 (85)	30 (65)	
Absolute risk difference (95% CI)	19.6% (1.9 to 37.2%)		0.030

PDT, photodynamic therapy.

## Discussion

Local tumour progression and sepsis secondary to biliary outflow obstruction has a significant impact on the survival of patients with BTC. Insertion of plastic or metal endoprostheses can alleviate biliary obstruction, but stent occlusion with recurrent cholangitis is a frequent problem. It follows that improved drainage with PDT may improve survival and this, supported by encouraging results from small randomised trials, was the hypothesis for the current randomised study. Unexpectedly, there was a significant survival detriment associated with PDT. This could be ascribed in part to less and later chemotherapy in the PDT arm, and potential late toxicity >1 month after PDT, as suggested by more early deaths associated with abnormal liver function.

Our findings were unexpected given the benefits of PDT reported elsewhere^8 9 14^ including a 12-month median survival in our own prior phase II PHOTOSTENT-01 trial.^12^ The study hypothesis was based on the promising data from Ortner *et al*^8^ and Zoepf *et al*.^9^ These studies were small (total patient numbers: 39 and 32 patients; with 20 and 16 patients receiving PDT in each study, respectively) and were therefore not sufficiently strong to define practice. Moreover, some of the studies included patients who remained jaundiced despite stenting and very few patients received systemic treatment—all factors associated with inferior survival. Two recent meta-analyses of the available data have suggested a benefit of PDT on survival on quality of life in BTC, but concluded that there was significant heterogeneity and the quality of evidence was low.^11 15^ Although the current study was closed on the advice of the Independent Data Monitoring Committee after 92 patients had been enrolled, it remains the largest prospective study evaluating the role of PDT in this setting.

The ABC-02 study which was conducted contemporaneously to PHOTOSTENT-02 randomised patients between gemcitabine and cisplatin with gemcitabine (CisGem).^16^ This study established CisGem as the international standard for advanced BTC (the median OS was improved from 8.1 to 11.7 months, p<0.001), but this was not standard practice at the time of the smaller German and UK PHOTOSTENT-01 studies. In PHOTOSTENT-02, patients randomised to PDT were more likely to be placed on surveillance following PDT, whereas those randomised to stenting alone were more likely to receive systemic chemotherapy and to receive chemotherapy sooner. Adjusting for whether patients had subsequent chemotherapy suggests that the reduced use of and delayed time to systemic treatment explained about 30% of the survival difference. Several other factors may also have played a role, in that patients randomised to PDT usually underwent an additional ERCP (with a greater risk of cholangitis) compared with the stenting alone group. To control for the effect of chemotherapy would require a study design that mandated standard chemotherapy but it is uncertain whether this would have been feasible or reversed the significant adverse outcome described in this study. In a randomised phase II trial of PDT±chemotherapy in patients with unresectable hilar cholangiocarcinoma, PDT plus the oral fluoropyrimidine, S-1 was well tolerated and associated with a significant improvement in OS compared with PDT alone (17 vs 8 months, p=0.005).^17^

In conclusion, in this multicentre study, patients with BTC who received PDT in addition to optimal stenting had a poorer OS than those with stenting alone, which was only partly explained by fewer patients in the PDT arm receiving palliative chemotherapy. Based on these results, we conclude that optimal stenting remains the treatment of choice for malignant biliary obstruction, and that the use of porfimer sodium PDT for this indication outside of clinical trials cannot be recommended.
